# A guide to the ecological limitations of phage therapy of bacterial biofilms: Is what's best for the phage best for the physician?

**DOI:** 10.1111/febs.70615

**Published:** 2026-06-08

**Authors:** Stephen T. Abedon

**Affiliations:** ^1^ Department of Microbiology The Ohio State University Mansfield OH USA

**Keywords:** antimicrobial resistance, bacteriophage therapy, biofilm, burst size, extracellular polymeric substance, latent period

## Abstract

Bacterial biofilms—structured communities of bacteria encased in self‐produced polymeric matrices—present formidable challenges in clinical medicine. The resistance of biofilms to conventional antibiotics stems from multiple factors. These include limited drug penetration and the presence of metabolically dormant bacteria that survive treatments, despite their retaining sensitivity under standard laboratory conditions. Bacteriophages (phages), the viruses that infect and kill bacteria, have emerged as promising alternatives or adjuncts to antibiotic therapy, including against bacterial biofilms. Phages, nonetheless, likely evolved to optimize especially their dissemination between spatially separated bacteria, including spatially separated biofilms, rather than to become specialists at eradicating all targeted bacteria from biofilms. By contrast, complete bacterial elimination from the body is the standard goal of antibacterial therapies. Considered here is how an understanding of these competing goals—virion dissemination vs. complete bacterial eradication—can inform our development of phage‐based anti‐biofilm therapies.

## Graphical abstract

Bacteriophages—viruses that kill bacteria—are promising weapons against bacterial biofilms: sessile communities notoriously resistant to antibiotics. Yet phages evolved not so much to eradicate bacteria as to obtain new bacteria to infect. That goal, however, may conflict with the physician's objective of complete bacterial clearance. The oversized phage virion symbolizes this tension: an evolutionarily refined predator hovering over a clinical problem it was not selected by nature to solve.

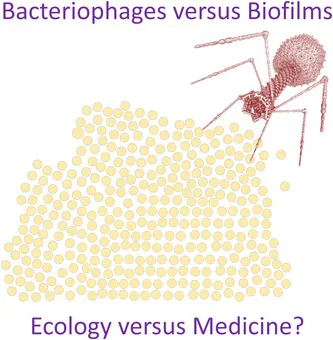

**Abbreviations**
EPS: Extracellular Polymeric Substances
MIC: Minimum Inhibitory Concentration
PAS: Phage‐Antibiotic Synergy

## Introduction

Resistance to antimicrobial therapies represents one of the most pressing challenges facing modern medicine [1, 2]. Among the factors contributing to treatment failures, bacterial biofilms play an outsized role [3, 4]. Biofilms are structured communities encased within a self‐produced matrix of extracellular polymeric substances (EPS) [5]. They typically are found adhered to surfaces such as abiotic materials like medical devices [6, 7, 8] or biotic tissues like heart valves [9]. EPS‐encased colonies of bacteria, however, can also exist as unattached aggregates [10], the latter being the case with *Pseudomonas aeruginosa* infecting cystic fibrosis lungs [11]. These EPS matrices are composed primarily of polysaccharides, proteins, extracellular DNA, and exoenzymes, where the latter can create “an external digestive system” [12]. Biofilms also create a microenvironment that can reduce antibiotic efficacy compared to planktonic (free‐floating) bacteria [13]. The clinical significance of biofilms therefore is substantial and, once established, these biofilm‐associated infections can prove remarkably difficult to fully eradicate [14, 15]. Furthermore, biofilms are highly diverse [10], undermining the prospect of one‐size‐fits‐all approaches to their clinical management.

One possible medical strategy in combating bacterial biofilms is the use of bacteriophages, or simply “phages,” that is, phage therapy [16, 17, 18, 19, 20, 21, 22]. Bacteriophages—literally devourers of bacteria—are viruses that specifically infect bacterial cells. Discovered independently by Frederick Twort in 1915 [23] and Félix d'Hérelle in 1917 [24], phages were recognized almost immediately for their antibacterial potential [25]. D'Hérelle himself pioneered phage therapy, treating dysentery patients as early as 1919, with promising results [26, 27]. The rise of broad‐spectrum antibiotics in the 1940s nonetheless led to phage therapy's abandonment in most of the Western world [26, 28], though it continued to be practiced and refined in Eastern Europe, particularly in Poland and at the Eliava Institute in Tbilisi, Georgia [29].

The mode of action for strictly lytic phages—the type most commonly considered for therapy—is the killing of bacteria [30]. These phages also display self‐amplification particularly at the site of a bacterial infection, as analogous to serving as an antibacterial Trojan horse [31], along with lysis of bacterial cells [30, 32, 33]. This combination of characteristics, including typically low phage toxicity [34] and low impact on microbiomes [35], makes phages attractive as “living antibiotics”. Numerous studies also have documented phage‐mediated biofilm reduction *in vitro*, though rarely achieving complete bacterial eradication [7, 36, 37, 38, 39]. This pattern of bacterial reduction without full bacterial clearance raises a fundamental question: Are phages, as products of natural selection, in fact optimized for the kind of complete bacterial eradication that physicians and their patients desire?

Here, where experimental evidence is available it is cited, but otherwise much of this discussion of phage‐biofilm interactions is built upon theoretical and conceptual work on this and related subjects that I have developed over the past nearly two decades [31, 40, 41, 42, 43, 44, 45, 46, 47, 48, 49, 50, 51, 52]. Much of this review is thus theoretical in nature but with a primary goal of providing a framework for thinking about phage‐biofilm interactions not just from medical but also ecological perspectives. Thus, is what is good for the phage always also best for the physician?

## Phages evolve to disseminate, not eradicate

To understand potential phage limitations as therapeutic agents, it is important to first consider the selective pressures that may have shaped phage evolution. Critically, in nature bacteria often exist not as homogeneous suspensions but as spatially structured populations, that is, as biofilms. Physical gaps—whether measured in micrometers or meters—can separate one biofilm from the next, creating a spatial feast‐or‐famine scenario for consumers of these biofilms. Within a clonal cluster of bacteria making up a biofilm, phage‐susceptible cells are abundant, potentially providing a local bonanza of bacterial hosts. Thus, individual phages ideally will establish a focus of infection within these clonal clusters of bacteria, where an initial phage infection results in a localized series of phage infections. Between these clusters of bacteria, however, can lie voids: regions devoid of susceptible bacteria, where phage virions must survive long enough to encounter the next cluster of potential hosts. A phage lineage succeeds only if, on average, each individual exploitation event—in this case a focus of biofilm infection—produces at least one progeny virion that similarly succeeds, and does so despite these voids [44, 46, 47, 50].

Phages thus need not eradicate all of the bacteria found within a biofilm to still be ecologically successful. Natural selection should instead act on biofilm‐exploiting phages to optimize their dissemination between biofilms (Fig. 1). That optimization certainly will involve phage infection of biofilm bacteria and, for lytic phages, subsequent lysis and release of new phage virions. But the goal will be to maximize the number of phage progeny that successfully disseminate to infect new biofilms rather than necessarily to maximize the killing of biofilm bacteria.

**Fig. 1:**
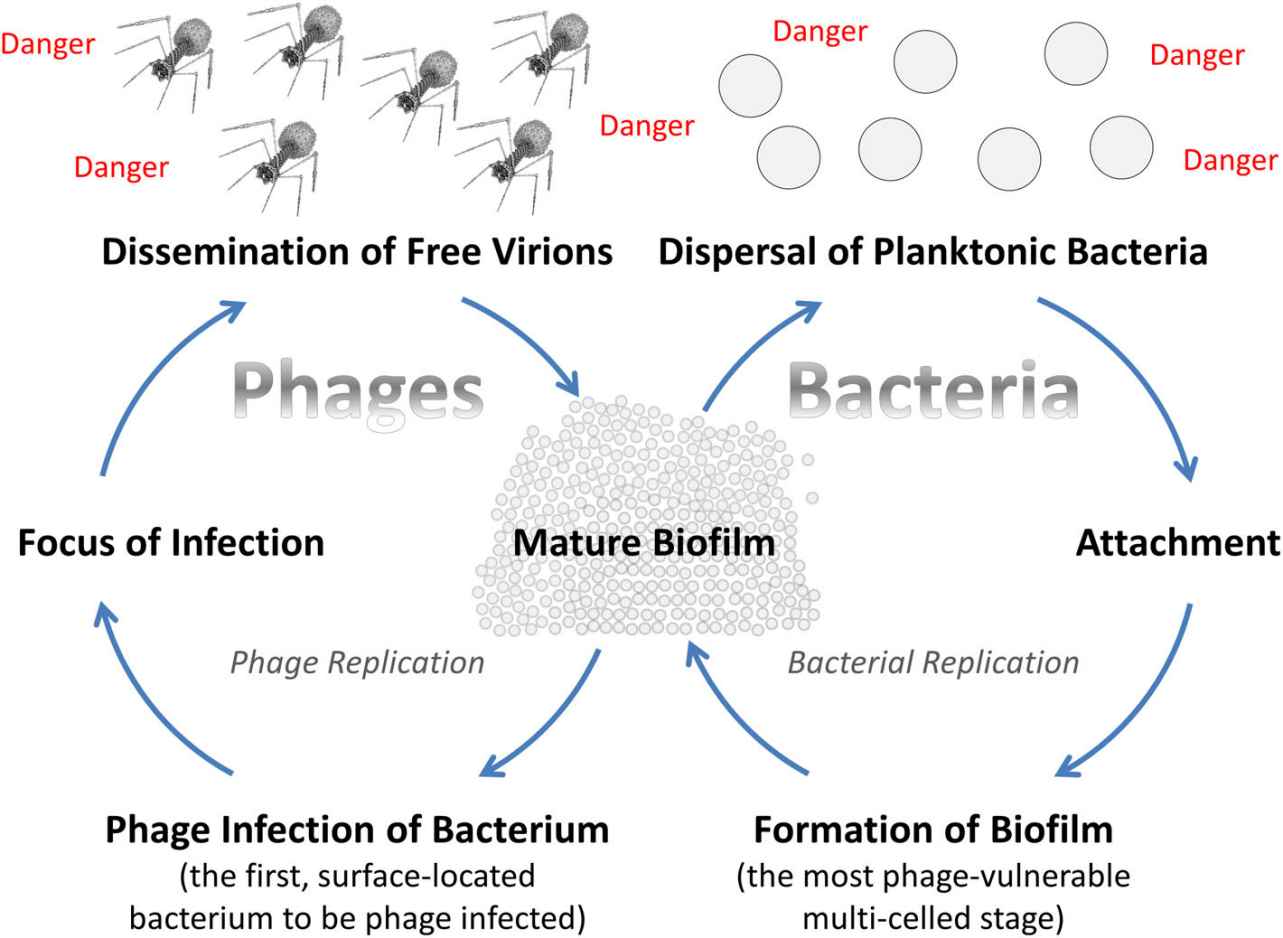
Parallel life histories: biofilm‐exploiting bacteriophages and biofilm bacteria. This figure illustrates the life cycles of bacteria (circles) forming biofilms (right) and phages (virions) exploiting those biofilms (left). The latter occurs once those biofilms have formed—indicated in the figure as “Mature biofilm” (middle), though encompassing also maturing biofilms. The top of the figure emphasizes that the dissemination/dispersal steps are not guaranteed to be successful (“Danger” for the microorganisms). Note that a focus of infection is defined as a localized area of phage population growth occurring in association with a bacterial biofilm, with parallels to the formation of phage plaques [122, 123, 124].

This phage dissemination imperative has several implications. First, phages must produce enough progeny virions to overcome the challenge of their finding rare, distant targets. Larger burst sizes—the number of virions released per lysed cell [32]—therefore should increase the probability that at least one virion will successfully disseminate. Producing more virions, however, takes time. This creates a trade‐off between expedience (lysing bacteria quickly to exploit neighboring bacteria before competitor phages arrive) and economy (lysing later to produce more disseminating progeny per bacterial infection) [53]. Which strategy is favored by natural selection should depend on environmental conditions, particularly the density and accessibility of susceptible bacteria [54].

Second, phages likely face diminishing returns as they penetrate deeper into biofilms. Bacteria on or near the surface of a biofilm tend to be healthier—more metabolically active and better nourished [55, 56, 57, 58]. Those surface bacteria therefore should be capable of supporting larger phage bursts [37, 59, 60]. Bacteria buried in biofilm interiors, conversely, experience nutrient limitations, accumulate waste products, and display reduced metabolic activity. Infecting these less metabolically active bacteria should yield fewer phage progeny while taking longer to produce those new virions. From an ecological perspective, deep biofilm penetration thus may offer only marginal benefits to phages: the relatively few additional virions generated by interior infections may take longer to produce and contribute less to virion dissemination success than infections of bacteria that are closer to biofilm surfaces and therefore closer to nutrients and oxygen [48]. The evolutionary question therefore is, should phages prioritize the infection of interior bacteria and should they do so if that capability pleiotropically affects their ability to infect surface bacteria?

To the extent that a bacterial biofilm or smaller bacterial aggregation does not possess regions of substantially reduced bacterial metabolic activity, or where barriers to phage or antibacterial penetration are otherwise absent, then this issue of diminishing returns should be less relevant ecologically, as well as medically. That is, the primary consideration in this review is phage activity against bacterial biofilms for which substantial barriers to phage‐mediated bacterial eradication exist, thus phages infecting recalcitrant bacterial biofilms. Absent such barriers, there should be little biofilm‐related concern for either phages or physicians.

Third, killing bacteria is only a *byproduct* of the replication cycle of lytic phages. Killing for killing's sake therefore likely does not provide a substantial fitness advantage to phages. It may even be detrimental given irreversible phage adsorption without subsequent virion production, such as due to infection of metabolically quiescent bacteria [60].

These considerations suggest that phages that have been shaped by billions of years of evolution in spatially structured environments need not be perfect killers. They need only be effective at infecting bacteria and then producing progeny virions that successfully disseminate to new bacteria that are themselves worth infecting. Furthermore, the framework developed here applies broadly, as metabolic gradients, diffusion barriers, and physiological heterogeneity—common features across biofilm types—create challenges for phages regardless of the specific biofilm architecture.

## Physicians seek bacterial eradication

Phage therapy differs fundamentally from the ecology of phages in nature. When a physician treats an infection, the goal ideally is complete bacterial eradication. Treatments which leave even a small number of bacteria behind—especially if those surviving bacteria are not subsequently eliminated by the body's immune system—represent clinically suboptimal outcomes. This is because these survivors can then reseed the bacterial infection, particularly once antibacterial treatment has been discontinued [15]. This is precisely the challenge when treating bacterial biofilms and the result is infection relapse. This relapse can occur even without bacterial evolution of resistance if treatment‐sensitive bacteria, such as biofilm‐associated persister cells [15], have not been completely eradicated. Biofilm infections consequently are an important contributor to infection relapse or may instead require chronic suppressive therapy, also known as maintenance therapy, to keep biofilm‐associated infections under control. There is an important distinction, in other words, between bacteriological cures, meaning bacterial eradication, and clinical cures, which are prone to infection relapse and require maintenance therapy [14].

For phage treatments of prosthetic joint infections, which typically are biofilm associated, the rate of remission determined via a meta‐analysis was calculated as high as 83%, implying 17% relapse, with equivalent outcomes reported in similar reviews [6]. Phage‐based treatments thus appear to be capable of achieving bacterial eradication, but not always. As a complication of such analyses, often for phage clinical studies it can be difficult to distinguish between phage‐mediated and antibiotic‐mediated positive results [61]—consequently, even with the achievement of bacteriological cure, it is not always easy to attribute that cure to phage action alone.

In natural environments, phage exploitation of biofilms likely begins with low phage numbers, such as a single virion, and this is especially so if phage dissemination to more‐distant biofilm targets is fraught. Consequently, to generate substantial phage numbers, that virion must then replicate, and for the sake of bacterial eradication, that replication must produce high numbers of virions [62]. Clinicians, by contrast, can administer phages already at high titers, ideally flooding a biofilm with virions; that is, unless phage pharmacokinetics are challenging. These high initial phage titers can in principle bypass the need for extensive local phage replication. Additionally, physicians can administer phages repeatedly [63], over days, weeks, and even months [61, 64, 65], sustaining what ideally will be inhibitory phage numbers over the course of treatments. Levin and colleagues, however, based on mathematical simulation of repeated phage and antibiotic dosing in a context of immune system impact on treating prosthetic joint infections [8], concluded that even multi‐dose phage treatments are unlikely to eradicate bacterial biofilms, with phages adding little to what antibiotics achieve alone.

Clinical outcomes with phage therapy thus remain inconsistent [22, 61, 66], and particularly so for established biofilm infections. This should be especially a concern with immunocompromised patients who can be inherently less able to clear remnants of bacterial infections on their own [67, 68, 69]. What might be the reason for why treatment outcomes are inconsistently effective? One answer may lie in the mismatch between phage evolutionary/ecological optimization and clinical needs. Phages that succeed in nature may possess traits that are suboptimal—or even counterproductive—for therapeutic biofilm eradication. Understanding these mismatches is essential for developing rational phage therapy strategies. The focus of this review is on how those suggested mismatches could manifest in terms of successful phage exploitation vs. successful phage treatment of bacterial biofilms.

## Contrasting ecological and therapeutic imperatives

Table 1 summarizes key differences between phage ecological goals and physician therapeutic goals. These contrasts illuminate specific areas where phage traits optimized for natural dissemination may conflict with clinical requirements. I examine several of these contrasts in detail below.

**Table 1:** Contrasting phage (ecological) and physician (therapeutic) perspectives.

Factor	Ecology	Therapy
Primary goals	Dissemination to new bacteria to infect	Eradication of bacterial pathogens
Objective of phage infections	Produce new virions	Kill the infected bacterium
Targeted bacteria	Healthiest, especially as found on or near biofilm surfaces	All, including especially those hardest to kill
Starting phage multiplicities	Very low (<< 1)	Can be high (> 1)
Penetration	Most accessible bacteria likely preferred for infection so penetration can be limited	Must penetrate to, and kill, all bacteria regardless of how deeply buried
Higher rates of virion adsorption	Probably useful for more effective dissemination (increased likelihood of successful adsorption)	Less need for higher rates, as lower rates can be compensated for by dosing with more phages
Importance of burst size	The more virions produced, the higher the potential for successful virion dissemination	Only matters if phages cannot be dosed in sufficient quantities (given challenging pharmacokinetics)
Importance of lysis	Means of initiating dissemination	Means of burrowing phages to non‐surface bacteria.
Importance of latent period	Shorter allows rapidity of local exploitation but at cost of burst size	Shorter could allow faster burrowing but also faster release of toxic lysis products
Utility of multiple dosing	More phages represent more competition to a phage lineage	Means of sustaining sufficient phage multiplicities over time
Evolution of bacterial resistance	Very minor impact on phage virion productivity assuming low frequency of resistance	Can result in failure to eradicate targeted bacteria: relapse with acquired resistance
Co‐treatment with inhibiting antibiotics	Antibiotics at standard inhibitory concentrations (≥ MIC*) generally are harmful to phage infections	Potential for additive interactions if bacterial targets differ between phages and antibiotics

* MIC = Minimum Inhibitory Concentration (of antibiotic on bacterial replication).

One theme is the utility of different phage life‐history traits, particularly adsorption rate, latent period length, and burst sizes. A general assumption is that faster bacterial acquisition, shorter infection durations, and greater virion production per infected bacterium, respectively, should be ideal toward achieving greater phage antibacterial virulence [30], particularly as a predictor of phage therapeutic effectiveness [70, 71, 72]. However, those phage characteristics may vary in their utility depending on context, for example, biofilm vs. planktonic bacteria, as well as dosing strategies, such as active vs. passive treatments. Experimentally disentangling the effects of latent period length from those of burst size furthermore is not straightforward, as the two traits are mechanistically coupled: manipulating one tends to also perturb the other.

### Biofilm surfaces vs. biofilm interiors

Phages logically encounter biofilm bacteria first at biofilm surfaces, with surfaces defined as regions to which phages, nutrients, and oxygen can most easily diffuse (Fig. 2). As noted, these surface bacteria should exhibit the highest metabolic activity within biofilms and thereby support larger burst sizes than less metabolically active bacteria. Phages that don't compromise their ability to robustly infect these healthier, surface bacteria [48] therefore should possess growth advantages over phages that do compromise those abilities, such as to emphasize instead their ability to robustly infect more interior bacteria. This is an assumption of trade‐offs in abilities—that a given phage cannot optimize everything, which also can be described as antagonistic pleiotropies. Thus, we can speculate that most or all phages more effectively infect those biofilm bacteria that are more readily reached, that is, “surface” bacteria.

**Fig. 2:**
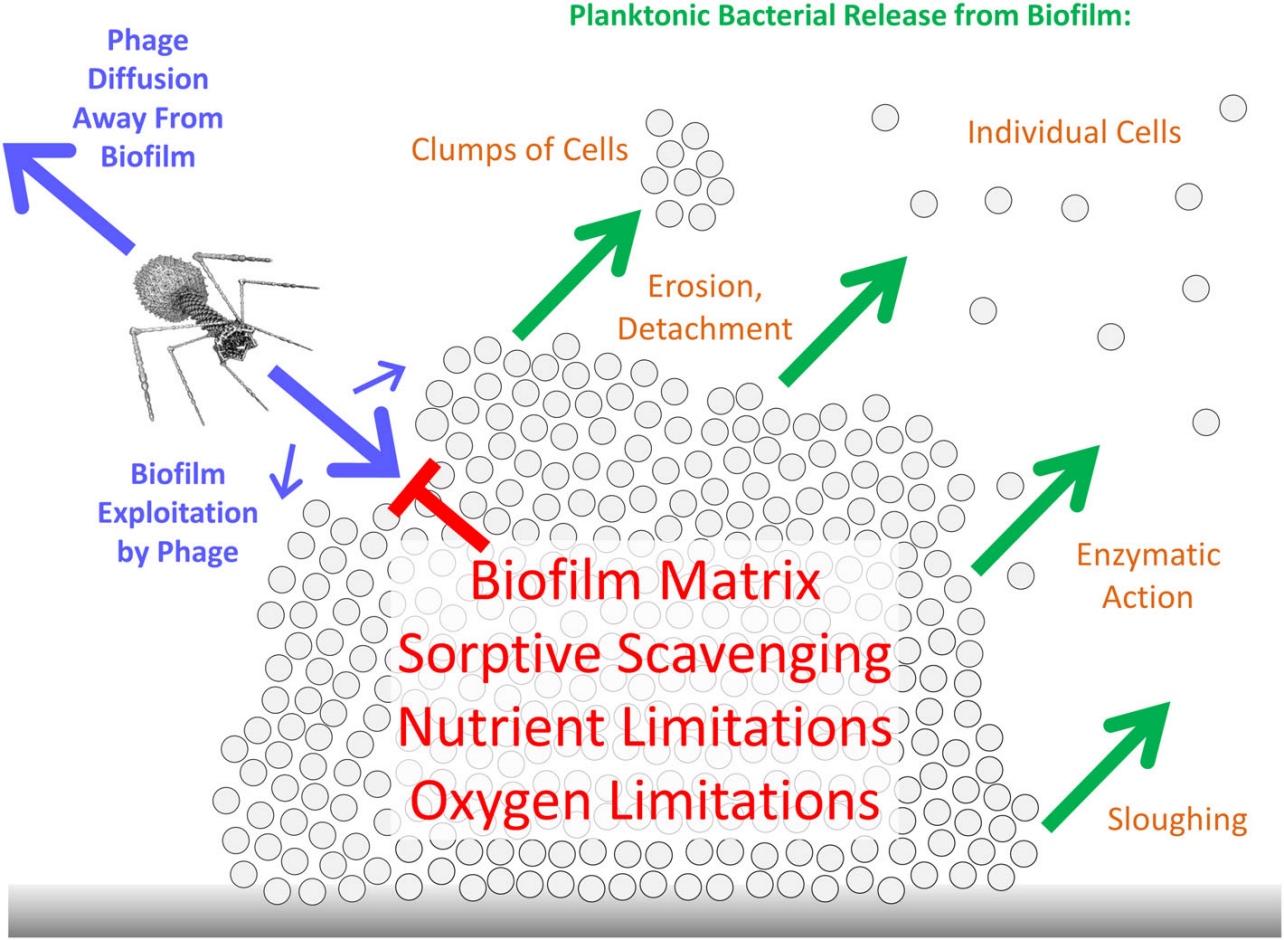
Limitations to phage penetration into bacterial biofilms (center, e.g., see [107] for review) and mechanisms of dissemination away from biofilms: phages (left) vs. bacteria (right). See Abedon [49] for consideration of bacterial dispersal mechanisms (grayer text and green arrows) along with the importance of these mechanisms as bacterial means of evading phage attack. In contrast to bacteria, many of which have an actively motile dispersion phase, phage virion dissemination is limited to diffusion. Bacteria within biofilms nonetheless are constrained, in their replication within biofilms, to binary fission—individual cells producing pairs of individual new cells. Phages, by contrast, have the potential to grow their populations while in association with biofilms by infecting multiple bacteria and producing many new phage virions (tens or even hundreds) per individual bacterial infection. Individual biofilm bacteria thus in many cases may be better adapted than phage virions to disseminating away from their biofilms of origin while phages, in contrast, may be more effective at producing large numbers of disseminating progeny per biofilm.

A phage bias toward infecting especially bacteria located nearer to biofilm surfaces, however, could be problematic therapeutically. Whether treating with phages or antibiotics, ultimately the most relevant bacteria are those that are hardest to kill: deep within biofilms, protected by mature EPS, and exhibiting reduced metabolic activity, which can confer phenotypic tolerance not just to antibiotics but likely also to phages [37, 52, 60]. Killing all the surface and mid‐layer bacteria, or even 99.9% while leaving some interior cells intact, is what leads to relapse of bacterial infections. Even so, such partial killing could presumably still result in ecological success in natural environments.

It is worth noting that phage‐encoded EPS degrading enzymes, that is, EPS depolymerases [73], can represent a means by which especially EPS barriers to phages within biofilms might be overcome. Though I share the hope of a utility for these enzymes as a part of comprehensive anti‐biofilm strategies, whether found associated with expressing phages or instead used as purified enzymes, EPS depolymerase enzymes nonetheless can possess significant shortcomings. These include (1) not all phages, including potentially useful therapeutic phages, naturally possess EPS depolymerases; (2) EPS depolymerases tend to be highly specific such that they do not necessarily work across all strains of a single bacterial species; (3) EPS can consist of multiple different polymers, potentially reducing the utility of a single enzyme activity even given matching of its specificity; (4) EPS can mature, increasing its potential to serve as a barrier to substantial phage penetration even when EPS depolymerases are present; and (5) there is an issue of successful systemic delivery of these enzymes in purified form given that they represent proteins that should be foreign to the body. Thus, EPS depolymerase enzymes likely do not represent a panacea for effecting full biofilm removal. Under appropriate circumstances, however, they may indeed be helpful in increasing biofilm removal, and perhaps particularly so if used in combination with additional biofilm‐removal strategies such as surgical debridement.

Biofilm interior bacteria thus are not expected to be thriving metabolically and should be better protected by EPS, especially as biofilms mature, potentially even given phage encoding of EPS depolymerase enzymes. Consequently, those interior bacteria are expected to be the key problems when battling biofilms in a medical setting, whether using phages or using antibiotics. Bacteria found on biofilm surfaces, by contrast, should be easier to access and, ecologically, should serve as more rewarding victims of phage attack [48]. Phages, that is, perhaps can be choosier in what bacteria they infect and then kill, but doctors cannot.

### Biofilm penetration: Marginal utility vs. therapeutic necessity

For phages to kill interior bacteria, virions must first reach those bacteria. Biofilm interiors, however, can be defined as places to which phages are unable to simply diffuse. Phage penetration to biofilm interiors thus likely requires more than just virion diffusion and likely also more than simply overcoming EPS barriers. One proposed model for such penetration—which I attribute to Elizabeth (Betty) Kutter—involves layer‐by‐layer bacterial lysis [42, 74]. Progeny virions released from more surface‐located bacteria may diffuse slightly further inward to infect the next bacterial layer, and so on. This process may be dubbed an active penetration of phages into biofilms [42] as effected not just by phage‐induced bacterial lysis but also by the production of new virions (auto dosing [75]).

This proposed layer‐by‐layer process is not necessarily without limitations. First, each lysis event is expected to release virions not just further into the biofilm but outward as well; that is, to effect virion dissemination away from the parental biofilm as well as lateral movement further across the surface of that biofilm [49]. Second, lysis in the vicinity of other bacteria should result in multiple adsorptions of individual bacteria, resulting in wastage of virions. This occurs because a single bacterium can support only one phage infection while also supporting many phage adsorptions. In some cases, multiple adsorptions might even interfere with virion production by that one phage infection [76, 77]. Third, the potential for infections to simply support phage replication should decline as phages move further into nutrient‐deprived biofilm interiors [60]. Fourth, as active penetration progresses, phages encounter EPS that may hinder further virion movement [78]. Layer‐by‐layer penetration thus can face multiple obstacles, encountered especially as phages reach more mature and nutrient‐deprived portions of biofilms.

EPS depolymerases might aid phages in this layer‐by‐layer penetration into a bacterial biofilm. Ecologically, however, it is uncertain to what extent this may be helpful, other than to allow phages to reach EPS‐encased bacteria more readily or faster. That is, if one takes as a premise that only phages that are capable of effectively infecting biofilm‐associated bacteria should be under consideration here, then what specific additional ecological utility may possession of EPS depolymerases provide, if infection is already a given? Nonetheless, keeping in mind the cautions already discussed, it is hoped that these enzymes may allow increased biofilm removal under certain circumstances at least.

Alternatively, and with or without the aid of EPS depolymerases, layer‐by‐layer penetration into biofilms might be effected by exogenously supplied phages. In this case, the primary requirement should be bacterial lysis to effect further phage penetration—potentially in combination with standard dosing rather than depending on *in situ* phage replication to supply required virions. By separating requirements for lysis from requirements for *in situ* virion production, one could employ anti‐biofilm strategies that are biased toward more effective and perhaps more rapid lysis. More rapid lysis, however, can be inconsistent with producing more virions per infection (next section). Nonetheless, it remains unclear whether lysis in combination with exogenous dosing alone can achieve deep penetration, as empirical data comparing these approaches remain sparse.

From an ecological standpoint, deep penetration strictly may not be crucial. If surface exploitation yields enough virions overall, with only marginal burst size benefits from penetrating ever deeper into biofilms, then phages need not evolve mechanisms for penetrating deeply into biofilm interiors, or at least not all phages. Therapeutically, however, reaching and then killing biofilm interior bacteria is essential, whatever the antibacterial agent used. Some phages, nevertheless, likely are better at this penetration than others, such as phages that are more effective at infecting physiologically older bacteria, as briefly discussed further below, or which produce and display EPS‐degrading enzymes on their virions.

Note that a different perspective on the utility of phage penetration into bacterial biofilms can be the idea that lysis of overlying bacteria could allow nutrients to more effectively diffuse to underlying bacteria, a perspective which I also attribute to Elizabeth Kutter. This is particularly given the removal of nutrient‐utilizing overlying bacteria or instead that the process of lysis itself could supply nutrients formerly tied up within intact, not‐yet‐lysed bacteria [79]. Given the release also of virions during this lysis process, we would expect that increased nutrient availability to formerly underlying bacteria could occur simultaneously with the initiation of new phage infections. This situation could be analogous to phage infection during the transition from stationary to log phase. As the productivity of phage infections is likely a function not just of nutrient availability but also ribosome availability within infected bacteria [59], I therefore speculate that increased nutrient availability that is roughly coincident with the start of phage infections still might result in lower phage infection productivity relative to infection of log‐phase bacteria. Still, from a medical perspective, such acquired nutrient availability might increase the potential for phages to kill, lyse, and even productively infect biofilm bacteria, especially if biofilms are being bathed with nutrient‐rich bodily fluids, such as blood.

### The importance and unimportance of burst size

Burst size—the number of progeny virions produced per infected bacterium—is a key phage life‐history trait. Larger burst sizes should be favored under many ecological conditions, such as to enhance dissemination between biofilms or simply to infect more bacteria locally per initially infected bacterium. However, achieving larger bursts typically requires longer latent periods, as assembling more virions demands more time and greater metabolic resources [54]. This trade‐off potentially creates a contradiction regarding phage choice for biofilm eradication.

An alternative, purely ecological perspective can be framed as the noted trade‐off between phage expedience (shorter latent period) and phage economy (larger burst size), which can affect the outcomes of direct phage‐to‐phage, exploitative competition. When multiple phages—even genetically similar ones—infect bacteria within the same biofilm, phages with shorter latent periods may be able to monopolize neighboring bacteria more rapidly as a local, exploitable resource by releasing virions sooner. This more rapid monopolization can be so even if it comes at the expense of achieving larger burst sizes. The result could be to drive phage latent periods shorter and burst sizes therefore smaller than might be optimal for subsequent dissemination were such within‐biofilm competition absent [54].

Independent of such within‐biofilm competition between different phage genotypes, Meneses *et al*. [38], in their systematic review of impacts of phages on *in vitro* biofilm, determined that in terms of *in vitro* biomass reduction, “phages with higher burst sizes and shorter latent periods seem to be the best candidates to control biofilms *in vitro*.” These correlations, while statistically strong for biofilm biomass removal, nonetheless were weaker for reductions in bacterial viable counts. In fact, no statistically significant impact was observed for differences in latent period for reductions in numbers of bacteria across the different studies reviewed. For the clinically important goal of bacterial eradication, greater burst sizes therefore could be more relevant than shorter latent periods. A concern with that conclusion, though, is that there are numerous caveats associated with how *in vitro* biofilm reduction assays may be conducted [51], potentially making conclusions reached across multiple studies less reliable.

A second issue is that burst size is not the only means by which phage numbers can be increased. Specifically, clinically the relevance of burst size should depend entirely on dosing strategy. If phages are supplied exogenously at high titers directly to bacterial biofilms, and can be re‐dosed as needed, then burst size can become nearly irrelevant: the physician can always add more virions. Indeed, Meneses *et al*. determined that adding more phages, at least *in vitro*, was also correlated with better reductions in both biofilm biomass and biofilm metabolic activity [38]. In contrast, if therapy relies on endogenous phage replication within a biofilm, that is, so‐called active treatment [42, 75, 80, 81], or indeed as a locally active treatment [82], then burst size should directly impact how deeply phages can penetrate into biofilms (see the previous section).

Very small burst sizes in particular—and certainly an absence of virion production entirely such as can be seen upon infecting stationary phase bacteria [60]—thus could limit effective phage penetration into biofilms, especially absent ongoing dosing with higher phage numbers. This distinction highlights again the difference between ecological constraints—where burst size matters greatly even if its magnitude ends up being a compromise—and therapeutic possibilities, where standard dosing in principle may in many cases be able to circumvent burst size limitations.

### The adsorption‐rate paradox

The rate at which individual phage virions adsorb bacteria—typically described by an adsorption rate constant [83, 84]—can affect ecological success. Higher adsorption rates in particular should increase the probability of effective virion dissemination: for phages moving between rare biofilms, higher‐probability adsorption upon encountering a susceptible target should be useful. Rapid adsorption nonetheless also means that a virion will more readily bind the first bacterium it encounters, even if that bacterium is found adjacent to a lysing bacterium. In a biofilm context, high‐adsorption phages thus may be less able to resist adsorbing immediately adjacent bacteria, as a manifestation of sorptive scavenging [49, 85, 86]. That could result in slower virion diffusion further into biofilm structures [87]. That is, such scavenging of virions risks “depleting the pool of freely diffusing viruses needed for propagation” [88]. Higher adsorption rates may also reduce the number of virions that are literally free to disseminate away from biofilms, as more virions instead adsorb locally.

In contrast to the ecological context where per‐virion adsorption rates are determined solely by a phage's genotype and circumstances, adsorption rates to individual bacteria can be modulated therapeutically by supplying more or fewer phages. That is, even phages displaying poor per‐virion adsorption could be boosted to higher overall rates of bacterial adsorption simply by dosing with higher phage titers [89]. Thus, disseminating phages in nature may or may not benefit from ever higher adsorption rate constants, though very slow adsorption rates may be particularly problematic ecologically for those phages. Therapeutically, by contrast, even slow adsorption is not necessarily detrimental, so long as this can be compensated for by dosing with more phages. Whether such adsorption‐rate fine‐tuning provides clinical benefits remains much more theoretically than empirically tested, however. A trend nonetheless seems to exist in the clinic in applying phages in greater numbers going from 2001 to 2021 [90].

### Resistance irrelevance vs. therapeutic failure

Most bacteria can readily evolve resistance to most phages [91]. For phage therapy, however, resistance evolution can be catastrophic. If even a small fraction of bacteria acquire resistance during treatment, while retaining the virulence of their wild‐type parents, then those bacterial mutants can repopulate an infection despite susceptible bacteria being cleared. This represents a relapse with acquired resistance, as distinct from infection relapse due to incomplete clearance of susceptible bacteria.

Resistance evolution, by contrast, can matter little ecologically, at least over shorter, that is, ecological time scales. This is because a phage lineage that infects a biofilm, produces progeny, and then successfully disseminates has achieved its evolutionary goal, and this can be the case even if phage resistance is selected for in the process of exploiting that biofilm. Just as killing of bacteria is not a phage's ecological priority, so too preventing evolution of phage resistance should not be a priority either, at least over the course of phage exploitation of a single bacterial biofilm. Importantly, what drives this disregard of resistance evolution is that phage‐resistant bacteria, as found within a population of otherwise genetically phage‐sensitive bacteria, should be quite rare, for example, on the order of only one in a 100 000 (10^−5^) bacteria or for many phage‐bacterial combinations, somewhat fewer [92].

Therapeutically, several strategies can mitigate resistance risk. Phage cocktails—mixtures of multiple phages with different receptor specificities—can be designed to reduce the probability that bacteria simultaneously evolve resistance to all treatment phages [91, 93]. Ecologically, however, introducing such competitor phages would not be to a phage's advantage. Alternatively, one can combine phages with non‐phage antibacterial treatments, such as antibiotics [36, 37, 39, 91]. Antibiotics, though, can themselves be ecologically detrimental to phages, as the following section considers.

### Utility vs. antagonism of antibiotics

The use of inhibitory concentrations of antibiotics in combination with phage treatments represents a near‐default clinical approach, given especially the ongoing use of antibiotics to control bacterial infections prior to and then during salvage therapies using phages [61]. Furthermore, much effort over the past decade [83, 94] has been devoted to what was described by Comeau *et al*. [95] as a phage‐antibiotic synergy (PAS), a concept which, as I argue [84], may trace back at least to the work of Hadas *et al*. [59] in 1997. PAS describes an improvement in antibacterial activity that is associated, as typically implemented, with the use of sub‐inhibitory concentrations of antibiotics. In clinical practice, however, antibiotics are typically administered at or above inhibitory concentrations, which limits the direct translational relevance of PAS as conventionally studied.

Chan *et al*. [96] by contrast presented an important clinical example of PAS using instead inhibitory concentrations of antibiotic in combination with phages. This involved an impact on bacterial evolution of resistance. The possibility that improved eradication of biofilms was involved as well, however, is consistent with *in vitro* results presented in the same study. Antibiotics thus are commonly used clinically in combination with phages, typically at or above minimum inhibitory concentrations, and there exists at least a potential for improved treatment results relative to the use of either approach alone, that is, just antibiotics or just phages.

Many antibiotics, especially if supplied instead at inhibitory concentrations, nonetheless can interfere with phage replication by reducing or altogether halting the bacterial metabolic activity that phages depend upon to successfully infect. This antagonistic effect recently has been described by Kaneko *et al*. [93] as “Anergy”, to contrast with synergy. From an ecological perspective, such antagonism is expected. Anything that slows bacterial metabolism—whether antibiotics, nutrient limitation, or environmental stress—can reduce a phage's burst size and extend its latent period. This would be antibiotic‐mediated antagonism of phage infection activity [97, 98]. Furthermore, antibiotic interference with the ability of phages to lyse bacteria could inhibit layer‐by‐layer phage penetration into biofilms (above).

Therapeutically, the situation nonetheless is more nuanced. While antibiotics may reduce phage infection activity, antibiotics also directly inhibit or kill bacteria, thereby contributing to bacterial clearance independent of phage action. Moreover, phages and antibiotics may be able to target bacteria complementarily [91], if not necessarily synergistically. Phages, for example, may be able to supply an alternative, self‐amplifying activity against bacteria that are less easily reached by antibiotics in sufficient concentrations [74, 99]. Antibiotics, by contrast, may be able to affect otherwise phage‐sensitive bacteria that phages are less able to reach, such as due to pharmacokinetic limitations on phage distribution, or otherwise are less able to successfully infect. This complementarity, however, can take time, as bacteria can transiently enter and leave susceptible states to both phages [52, 100, 101] and antibiotics [102, 103, 104].

Overall, phage‐antibiotic combination therapy represents a rational and indeed often clinically inevitable strategy [61], even if it results in only additive interactions or instead “facilitation” [105] rather than synergistic effects. Outcomes nonetheless will depend on antibiotic class, dosing levels, and treatment sequence, along with specific infection context [106]. These are issues that may be explored *in vitro* based on what are known as time‐kill curves in the antibiotics literature [30], and time‐kill approaches can be applied to phages or phage‐antibiotic combinations as well as to anti‐biofilm activities [106]. The key insight nonetheless is that antibiotics inhibiting phage replication—an ecological liability—may be acceptable clinically if the combined antibacterial effects exceed what either agent can achieve alone.

## Compensating for ecological optimization

Recognizing that phages have evolved for ecological success rather than for therapeutic bacterial eradication suggests several strategies to enhance clinical efficacy, many of which are suggested by Pires *et al*. [107]:

1. Optimize Dosing Regimens: Use high phage titers to reduce dependence on local phage replication. Repeat dosing to ensure ongoing sufficient virion supply.
2. Select Phages for Therapeutic Traits: Rather than choosing phages based solely on host range [33, 92] or even for just rapid population growth [30], prioritize traits that could be more relevant to biofilm eradication. These might include (2a) shorter latent periods for rapid action, but also (2b) production of EPS depolymerase enzymes, though the latter, as noted, are often bacterial strain‐specific in their ability to degrade particular EPS components [36, 37, 73, 108, 109]. In addition, (2c) an ability to lyse bacteria that are less physiologically healthy could be helpful [110, 111, 112]. Furthermore, though not specific to biofilm treatment, it is possible (2d) to select phages that are able to pleiotropically steer phage‐resistant bacterial traits toward desirable outcomes [113, 114, 115, 116, 117], which in at least one case has been found to reduce biofilm formation potential [118].
3. Employ Phage Cocktails: Mixtures of phages with complementary properties can be used toward either anti‐biofilm diversity (previous point) or as a hedge against resistance evolution. In principle, such phage combinations might be applied in a logical order rather than simultaneously; for example, EPS depolymerizing phages that are followed by phages with shorter latent periods, that are followed in turn by phages that are better suited to infecting and lysing physiologically older bacteria. Or phages might be engineered to display all three properties, such as an EPS depolymerase‐producing phage T7 [119].
4. Combine Phages with Antibiotics: Despite potential anti‐phage antagonisms, phage‐antibiotic combinations can be clinically superior to monotherapies. Temporal sequence may again matter, however: initiating with phages to disrupt biofilm structure, perhaps particularly in terms of their abilities to lyse bacteria, but then followed by antibiotics to clear exposed bacteria could better exploit each agent's strengths [36, 120]. Furthermore, biofilms can be partially disrupted mechanically—such as via surgical debridement, a common practice in the treatment of chronically infected wounds—to reduce some of the burden on phages.
5. Combine Phages with Adjuvants: These can include purified enzymes that degrade EPS, such as dispersin B and alginate lyase [121]. Again, however, the specificity of these agents may be limiting. Also, not all potentially useful adjuvants have been approved for human use, nor in combination with phages, representing a complication to regulatory approval of specific phage‐based treatments. See Kosznik‐Kwasnicka *et al*. [94], however, for review of combining natural substances such as essential oils, but also peptide bacteriocins or nanoparticles, with phages to improve antibacterial efficacy, including against biofilms.

## Conclusions and perspectives

Bacteriophages offer considerable promise as antibacterial agents, including against biofilm‐associated infections that resist conventional antibiotics. Not all biofilm‐exploiting phages, however, are necessarily optimized for the kind of complete bacterial eradication—whether 100% phage mediated or in combination with immune system action—that clinical medicine seeks. Instead, phage evolution likely has prioritized improving dissemination between spatially separated bacteria rather than the killing of every possible susceptible bacterium. That is, phages likely strive for a goal of only “good enough” bacterial killing, so as to produce and then disperse simply adequate numbers of new virions. The more phages that successfully disseminate the better, but that isn't necessarily accomplished by killing every possible bacterium locally.

This fundamental tension—between ecological optimization for dissemination and therapeutic requirements for eradication—manifests in multiple ways. Phages may preferentially target healthy, surface‐localized bacteria while somewhat neglecting the nutrient‐starved, deeply embedded bacteria that physicians most need to eliminate. Phage penetration into biofilms may be limited by various barriers while also not representing an ecological necessity. Phage life‐history traits such as latent periods and burst sizes, shaped by competition and dissemination demands, may not align with therapeutic optima against problematic biofilms. And bacterial resistance evolution, tolerable ecologically especially over shorter timescales, can rapidly derail therapeutic interventions, but not phage dissemination away from biofilms to find new bacteria to infect.

Yet this ecological–clinical mismatch is not insurmountable and recognizing it allows us to design compensating therapeutic strategies. High‐titer dosing bypasses ecological scarcity. Repeated administration provides continuous virion supply, ideally overcoming penetration barriers. Phage cocktails and other combination therapies hedge against resistance but also could exploit complementary anti‐biofilm mechanisms.

The key insight is that optimizing phage therapy may require more than identifying phages with just good ecological fitness, such as high plaquing efficiency, rapid bacterial killing in broth, or broad host ranges. It requires asking also: Is what's best for the phage always best for the physician? Often, the answer may be, “not entirely”. By understanding where phage ecological goals diverge from therapeutic needs, we can bridge that gap through thoughtful design, to better harness the power of bacterial viruses to combat some of medicine's most challenging infections.

## Author contributions

STA conceived of and authored this analysis.

## Conflict of interest

S.T.A. has consulted for and served on advisory boards for companies with phage therapy interests, holds an equity stake in a number of these companies, and maintains the websites phage.org and phage‐therapy.org. No additional competing financial interests exist. The text presented represents the perspectives of the author alone, and no outside help was received in its writing.
